# Portable Point-of-Care Uric Acid Detection System with Cloud-Based Data Analysis and Patient Monitoring

**DOI:** 10.3390/bios16020076

**Published:** 2026-01-27

**Authors:** Yardnapar Parcharoen, Pratya Phetkate, Kanon Jatuworapruk, Calin Trif, Chiravoot Pechyen

**Affiliations:** 1 Chulabhorn International College of Medicine, Thammasat University, Pathum Thani 12120, Thailand; yardnapa@tu.ac.th (Y.P.); pratya@tu.ac.th (P.P.); 2 Department of Medicine, Faculty of Medicine, Thammasat University, Pathum Thani 12120, Thailand; kanon@tu.ac.th; 3 Department of Materials Technology and Textile, Faculty of Science and Technology, Thammasat University, Pathum Thani 12120, Thailand; 4 Thammasat University Center of Excellence in Modern Technology and Advanced Manufacturing for Medical Innovation, Thammasat University, Pathum Thani 12120, Thailand

**Keywords:** uric acid, UV–Vis spectrophotometry, point-of-care testing, gout, urine screening

## Abstract

Uric acid is closely related to diseases such as gout, kidney failure, and metabolic disorders. A conventional method for measuring uric acid over 24 h is time intensive and cumbersome for patients who have to take samples to the hospital. At present, hospitals use only laboratory instruments to determine 24-h uric acid concentrations in the urine. This study presents the proof-of-concept of a portable point-of-care tool called Uricia, designed to improve the quality of life of patients monitoring uric acid. Spectrophotometry was performed at a fixed wavelength of 295 nm. The urine sample contained within the cuvette absorbs ultraviolet light, with uric acid specifically responsible for this absorption, thereby allowing the device to measure its concentration. An internal calibration algorithm was used to accommodate the nonlinear optical response of Uricia and was calibrated to a benchtop GENESYS 10S UV–Vis spectrophotometer. The experiments further evaluated potential urinary interferences, revealing that while most constituents had minimal impact, ascorbic acid demonstrated the highest interference, contributing up to 15% of the total signal at high physiological concentrations. This device and the corresponding spectrophotometry method revealed that high concentrations of uric acid precipitated insoluble crystals. A dilution set to an alkali solution vial to be premixed and dissolve the uric acid crystals was added, increasing the detection window to 10 mg/dL, with an LOD of 0.0232 mg/dL and LOQ of 0.0702 mg/dL. Cloud-based data measurement enables spot analysis, which is meant to provide insight into patient status development. These results validated the technical architecture of a controlled matrix for measuring uric acid.

## 1. Introduction

Gout, a severe form of inflammatory arthritis, is characterized by the accumulation of monosodium urate (MSU) crystals in the joints and supporting tissues. It is more common in males than in females and is reported to occur at a 3–6% prevalence in men and 1–3% in women in wealthy countries [1]. The risk of developing gout is higher in old age, especially after 50 years. Men tend to have a higher prevalence than women in their lifetime, with women often developing gout later in life by the time after menopause [2]. MSU crystals are directly related to chronic hyperuricemia, a metabolic condition associated with elevated serum uric acid (SUA) [3,4]. Timely and effective treatment of gout is based on the management of SUA levels. Routine monitoring of SUA is important for directing the treatment of urate-lowering therapy (ULT), the predominant objective of which is the dissolution of current MSU crystals, inhibition of the formation of new MSU crystals, and reduction in the rate and intensity of disease flares [5].

In addition to its direct involvement in the pathogenesis of gout, hyperuricemia has also recently been shown to add a major contribution significantly to a range of systemic conditions. Research indicates associations of high SUA levels with hypertension, metabolic syndrome, insulin resistance, chronic kidney disease, and cardiovascular disease at both epidemiological and clinical level [5,6,7]. Details of their specific mechanisms remain to be elucidated, but it appears from the available literature that hyperuricemia can induce endothelial dysfunction, oxidative stress, inflammation, and vascular damage to the renal microvasculature [8,9]. These data illustrate the wider clinical importance of uric acid imbalance, particularly from the perspective of global patient status and the need for coordinated management of hyperuricemia.

As renal uric acid excretion is associated with overall urate homeostasis [6,10,11] and approximately 70% of urate elimination is performed by the kidneys, accurate characterization of urinary uric acid excretion is necessary for both the diagnosis and intervention of gout. Quantifying urinary uric acid allows for differentiation between people who produce too much or not enough uric acid and guides the pharmacological treatment options used. Excess uric acid production may be improved in the presence of xanthine oxidase inhibitors, whereas patients with higher uric acid excretion might do better with uricosuric agents or those targeted for urine alkalization [4,12,13].

Urinary uric acid excretion is of considerable clinical relevance; however, its accurate evaluation in everyday clinical practice is limited [14]. Historically, the gold standard for this evaluation has required 24 h of urine collection and subsequent laboratory review at a certified centralized laboratory. However, this method has several drawbacks. Urine collection over 24 h is an exhausting process for patients, which also entails strict observation of sample collection, and incomplete or incorrect urine samples are frequently collected. In addition, infrastructure challenges during sample transportation and centralized laboratory analysis delay the results from being collected by a considerable margin, and the application of these results in clinical decisions is delayed [15,16].

Established analytical methods for urinary uric acid detection are represented by enzymatic assays and High-Performance Liquid Chromatography (HPLC), which serve as the gold standards for clinical accuracy. Enzymatic uricase-based assays rely on the oxidation of uric acid to allantoin and hydrogen peroxide, which subsequently react with chromogenic substrates to produce a measurable color change. While highly specific and suitable for high-throughput automation, they are limited by potential enzyme instability, complex incubation steps, and susceptibility to interference from substances such as ascorbate [17,18]. Alternatively, HPLC utilizing reversed-phase separation and UV detection at ~284 nm offers superior accuracy and multi-analyte specificity, particularly when combined with deproteinization techniques [19,20]. However, HPLC’s reliance on expensive instrumentation, technical expertise, and time-intensive sample preparation renders it impractical for rapid, decentralized testing.

Over the last decade, point-of-care testing (POCT) has become increasingly popular as a promising strategy for improving diagnostic access to the most common and time-efficient form of assessment. Such devices allow for rapid, onsite determination of all these analytes, removing the requirement for centralized, laboratory-based testing (at the point-of-care) and shortening its duration to obtain clinically relevant results. With the particular point of interest in uric acid assessment, POCT devices may improve rapid diagnosis, rapid adaptation to ULT management, and personalized care approaches. Spectrophotometric and electrochemical approaches have started to address the need for accessible point-of-care monitoring. Direct UV spectrophotometry (~290–300 nm) provides a simple, reagent-minimal, and cost-effective method that is adaptable to portable devices, although it necessitates careful calibration to mitigate spectral interferences from co-absorbing urine constituents [21]. Alternatively, emerging biosensors employing uricase immobilized on nanomaterials, such as polyaniline-polypyrrole films or ZnS nanostructures, demonstrate ultrasensitivity and real-time detection capabilities suitable for various biofluids. Despite their promise, these biosensor methods face ongoing challenges regarding enzyme immobilization stability and response time optimization, which currently limits their widespread commercial application [22,23,24]. In addition, this trend has the added disadvantage that the most commonly deployed POCT devices tend to have complex operation protocols, cost challenges, and a lack of efficient data management capabilities [25,26,27].

The current study investigated the Uricia prototype, an innovative point-of-care testing device developed for the rapid and accurate measurement of urinary uric acid content. Uricia integrated a low-cost and advanced analytical method to eliminate some of the deficiencies present in other POCT systems in their performance through its streamlined operation [28]. Unlike common enzymatic test strips, which provide semi-quantitative or qualitative results and are sensitive to humidity, Uricia offers quantitative digital data comparable to benchtop instruments. Furthermore, by utilizing low-cost UV-LED technology rather than the expensive monochromators found in handheld spectrometers, Uricia maintains a low bill of materials while offering cloud connectivity that standalone devices lack. Furthermore, Uricia distinguishes itself from standalone handheld spectrometers by incorporating an HIPAA-compliant cloud architecture with machine-learning capabilities. This connectivity allows the device to move beyond simple spot measurements, utilizing algorithms to estimate 24-h uric acid excretion trends, thereby bridging the gap between convenient home monitoring and clinically actionable data management. Through a methodical comparison of its analytical accuracy, precision, and robustness to a validated lab method on the relevant uric acid levels and clinical scenarios, this study attempted to solve the knowledge gap so that professionals could have access to a better and trustful method designed to provide reliable long-term monitoring and screening of patients with gout and hyperuricemia. The results of this study could improve the quality of care for health professionals in making more informed and prompt clinical decisions to treat a population with gout, for which the results can lead to better patient outcomes.

## 2. Materials and Methods

### 2.1. Materials

All reagents used in this study were of analytical grade. Uric acid (CAS 69-93-2), artificial urine (UNSPSC 12352202), glucose (CAS 50-99-7), ascorbic acid (CAS 50-81-7), albumin (CAS 9006-59-1), and creatinine (CAS 60-27-5) were obtained from Sigma–Aldrich (Bangkok, Thailand). Deionized distilled water (DDW) was obtained using a Milli-Q purification system (Millipore Sigma, Bangkok, Thailand). Sodium hydroxide (NaOH; CAS 1310-73-2) was purchased from Fisher Scientific (Bangkok, Thailand).

### 2.2. Equipment

#### 2.2.1. Conventional UV–Vis Spectrophotometer

Spectral measurements were performed using a GENESYS 10S UV–Vis spectrophotometer (Thermo Fisher Scientific, Bangkok, Thailand) operating between 195 and 1100 nm with 1 nm resolution. The benchtop device was used to characterize the absorbance profiles of uric acid and potential interferents and to provide reference measurements for validating the Uricia device.

#### 2.2.2. Uricia Point-of-Care Device

The portable Uricia device incorporates a 295 nm LED light source (Model HOUKEM-3W-295-6565, HOUKEM, Dongguan, China), a multispectral sensor (AS7343, OSRAM, Styria, Austria), and standard 1.5 mL semi-micro UV–Vis cuvettes (BRAND GMBH, Baden-Württemberg, Germany). Designed for point-of-care and laboratory settings, the device was compact, cost-effective, and powered by a rechargeable 9 V/1000 mAh lithium battery (Hi-Watt Battery Industry, Guangzhou, China). Bluetooth Classic 2.0 (DSD TECH, Shenzhen, China) enables wireless data transmission to an Android application and subsequent cloud integration.

**Figure 1:**
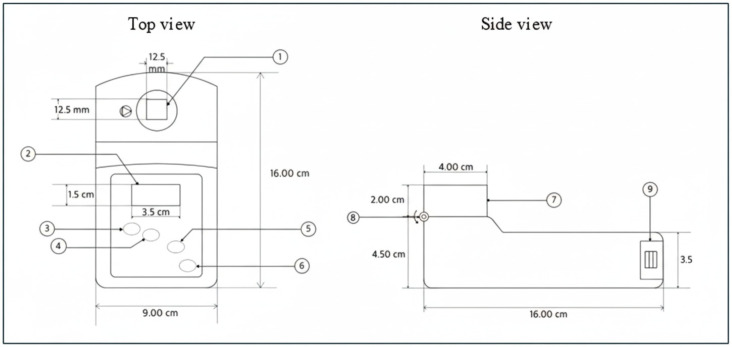
The portable UV measurement device was specifically designed for 295 nm readings, highlighting its compact and ergonomic design suitable for diverse testing scenarios. The device consists of a cuvette slot (1), display screen (2), uric acid measurement button for female users (3), uric acid measurement button for male users (4), general laboratory measurement button (5), power on/off button (6), device lid (7), hinge joint (8), and battery compartment (9).

**Figure 2:**
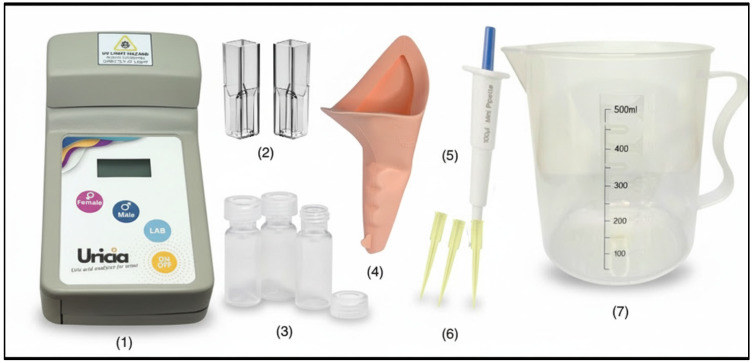
The portable UV measurement device set consisted of the Uricia device (1), 1.5 mL disposable UV–Vis cuvette (2), 2 mL Polypropylene Vials (3), silicone tube assists (4), 100 µL Mini Fixed Volume Micro Pipette (5), pipette tips (6), and 500 mL beaker (7).

Figure 1 and Figure 2 illustrate the Uricia device and its accessory components, respectively. The major features include a cuvette slot (1), display screen (2), gender-specific uric acid measurement buttons (3–4), general laboratory measurement button (5), power switch (6), protective lid (7), hinge assembly (8), and battery compartment (9). Gender-specific buttons allow optional tagging of readings for downstream demographic analysis. The measurement kit consisted of disposable cuvettes, polypropylene sample vials, silicone tubing for urine collection assistance, a 100 µL fixed-volume micropipette, pipette tips, and a 500 mL beaker for general handling.

### 2.3. Methods

#### 2.3.1. Uric Acid in Artificial Urine and DDW

Uric acid solutions (0, 1.25, 2.5, 5, 10, and 20 mg/dL) were prepared using artificial urine and DDW. The mass was measured using an analytical balance (Mettler Toledo, Bangkok, Thailand). Optical density (OD) at 295 nm was measured using both the GENESYS 10S spectrophotometer and the Uricia device.

#### 2.3.2. Uric Acid in Artificial Urine with NaOH Adjustments

To improve the solubility at higher concentrations, solutions were prepared in artificial urine and treated with 3% (*v*/*v*) 1 M NaOH. The concentrations matched the DDW test range. OD readings were collected under identical measurement conditions.

#### 2.3.3. UV–Vis Profiling of Uric Acid and Interferents

Solutions containing uric acid (5 mg/dL), glucose (250 mg/dL), ascorbic acid (2.5 mg/dL, 50 mg/dL), albumin (0.5 g/dL), and creatinine (1 mg/dL) were analyzed individually and in mixtures to assess spectral interference effects.

#### 2.3.4. Calibration, Data Processing, and Mobile Application

Before each measurement sequence, the Uricia device was calibrated using DDW as a blank. During calibration, the system recorded the baseline OD and adjusted the internal calibration curve accordingly. The calibration parameters were stored locally and in the cloud. A control standard of known uric acid (UA) concentration was analyzed periodically to verify accuracy, and recalibration was conducted if the deviations exceeded the acceptable limits.

The OD data were transmitted via Bluetooth to the Uricia Android application, which performed preprocessing (error checking, outlier detection, and OD-to-concentration conversion using the built-in calibration model). The data were then encrypted and uploaded via the Web Bluetooth API to the HIPAA-compliant Uricia Cloud server for secure storage and downstream analytics.

### 2.4. Statistical Analysis

Descriptive statistics (mean, SD, and variance) were computed using Python 3.11.6. One-way analysis of variance (ANOVA) was used to assess the concentration effects within each device. Two-way ANOVA was used to evaluate device, concentration, and interaction effects. Regression models were developed to characterize the OD–concentration relationship. Data visualization was performed using Microsoft Excel.

## 3. Results and Discussion

### 3.1. Uric Acid Measurement in Artificial Urine

**Figure 3:**
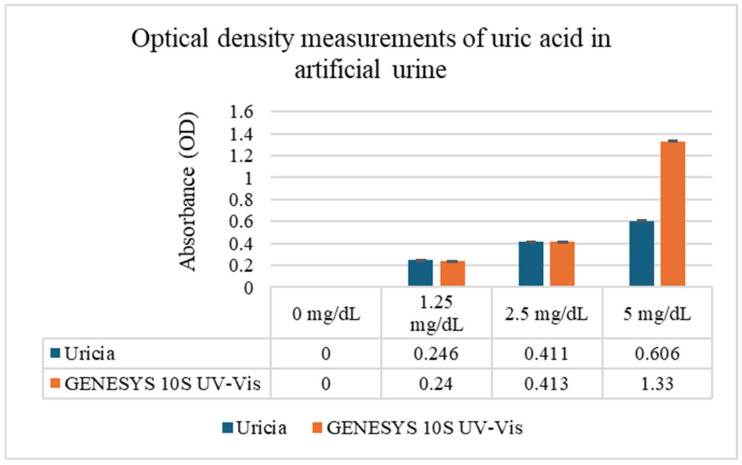
Comparison of uric acid solution analyses using the Urica device and GENESYS 10S UV–Vis Spectrophotometer at various concentrations (SD < 0.01).

Uric acid solutions prepared in artificial urine (0–20 mg/dL) were evaluated using Uricia and the GENESYS 10S UV–Vis spectrophotometer (Figure 3). At lower concentrations (0–5 mg/dL), the two devices generated closely matching optical density (OD) measurements, demonstrating that the simplified optical architecture of the Uricia device can be reliably used for quantification under conditions of complete UA solubility. However, it is necessary to note that there was a visible precipitation from uric acid that did not fully dissolve, resulting in saturated measurements at concentrations of 10 and 20 mg/dL. However, these solubility limitations arise from uric acid chemistry and are not specific to the type of device.

The results from the two systems are compared in Figure 3. The high level of agreement at soluble concentrations indicates that Uricia is appropriate for point-of-care environments, where fast screening is emphasized and access to benchtop equipment is limited. Solubility parameters of uric acid exceeding ~5 mg/dL often cause absorbance to plateau, even with laboratory spectrophotometer tests, leading to a lack of analytical range without sample dilution.

To quantify the concentration–response relationship, we used four uric acid concentrations (0, 1.25, 2.5, and 5 mg/dL) in eight measurement cells over two devices with triplicate readings. OD increase with concentration was confirmed by means of one-way ANOVA carried out for each device (Uricia: F = 1.5 × 10^9^, *p* ≈ 2.6 × 10^−23^; Genesis 10S: F = 3.2 × 10^9^, *p* ≈ 1.2 × 10^−24^). The residual sum of squares is almost zero, which suggests excellent internal precision of the device. The two-way ANOVA indicated that the device, concentration, and interaction had significant effects, showing that the difference between the two devices is not one constant offset but instead depends on concentration, typically seen with portable and benchtop optical comparisons. The rapid rise in OD to 5 mg/dL (maximum at saturation) necessitated the calibration of this linear region.

### 3.2. Uric Acid Measurement in Artificial Urine with NaOH Adjustment

**Figure 4:**
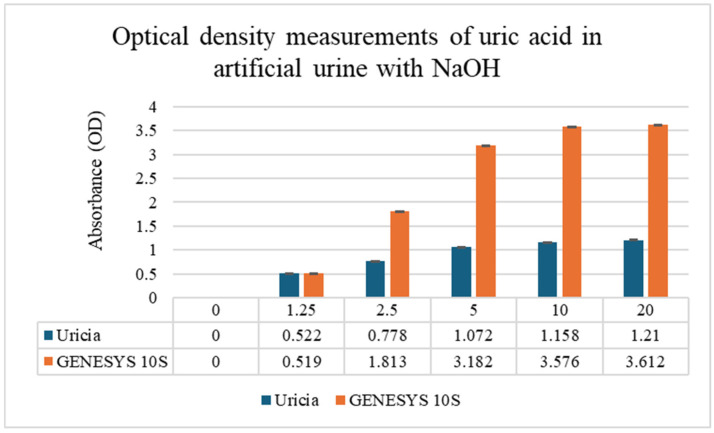
Uric acid measurements in artificial urine with NaOH adjustment demonstrated improved solubility and device performance (SD < 0.01).

To overcome solubility limitations at higher concentrations, 3% NaOH was added to the artificial urine to obtain uric acid solutions at higher concentrations. Mild alkalinization increased crystal dissolution while boosting measurable OD values. The concentration of 3% (*v*/*v*) 1 M NaOH was empirically optimized. This level provided sufficient alkalinity to solubilize uric acid crystals up to 10 mg/dL without reaching a threshold that would induce the precipitation of other urinary solutes or significantly alter the optical properties of the matrix. GENESYS 10S supplied higher absolute OD values (given its fixed optical path length, stable lamp output, and more sensitive detector); however, with respect to the Uricia device, they were proportional and reproducible (Figure 4; GENESYS 10S: R^2^ = 0.98; Uricia: R^2^ = 0.90). The Uricia usable upper limit, after alkalinization, increased from 5 mg/dL to 10 mg/dL with no distortion of the calibration curve, which is in line with other reports using alkaline media with strong absorbance of uric acid between 290 and 300 nm [21]. Because the OD values derived specifically from portable and benchtop devices are intrinsically different based on the optical geometry, calibrated concentration estimates require conversion using device-dependent standard curves. The Uricia software embeds this transformation, providing a cubic regression model to calculate precise concentration estimates from real-time data. Together, these results demonstrate that Uricia combined with NaOH-assisted solubilization can measure uric acid across a clinically relevant range with reliability in agreement with traditional UV–Vis methods [21,29].

### 3.3. UV–Vis Spectrophotometric Profiling

**Figure 5:**
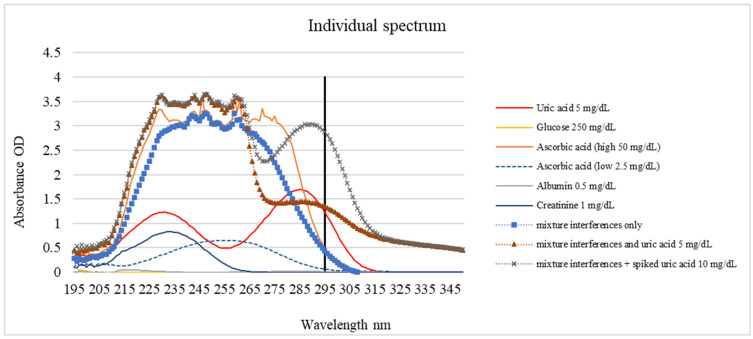
UV–Vis spectrophotometric profiling of glucose, ascorbic acid, albumin, creatinine, and uric acid.

Spectral profiling was performed to investigate the interference generated by the primary urinary constituents: glucose, ascorbic acid, albumin, and creatinine. The absorption characteristics of each compound at 200–350 nm are shown in Figure 5 and Table 1. Uric acid exhibited a dominant peak near 295 nm and was used as the basis for selective detection. Due to the lack of conjugated bonds, glucose exhibited negligible absorbance, albumin and creatinine displayed peaks below 250 nm corresponding to peptide and heterocyclic structures, and ascorbic acid absorbed strongly between 240 and 280 nm with diminishing intensity toward 295 nm. Although the absorption peak of uric acid occurs at 285 nm (Table 1), a wavelength of 295 nm was specifically selected for the Uricia device. As shown in Figure 5, potential interferents, such as ascorbic acid and proteins, exhibit strong absorbance below 290 nm. By measuring at an off-peak wavelength of 295 nm, the device minimized spectral interference from the urine matrix while maintaining sufficient sensitivity for uric acid detection.

Mixture spectra showed that the uric acid peak remained discernible even when all interferents were present simultaneously, maintaining a clear maximum between 250 and 265 nm, depending on the concentration.

While a detection wavelength of 295 nm was selected to reduce spectral overlap, it does not entirely eliminate interference. As shown in Figure 5, ascorbic acid exhibits a broad absorption band that tails into the 295 nm region. At a concentration of 50 mg/dL, which is higher than the mean physiological concentration observed in patients taking vit. C supplementation (37 mg/dL) [30], ascorbic acid contributed approximately 15.27% to the total signal in the mixed samples. While the calibration algorithm can account for the baseline levels of common interferents, users should be aware that high concentrations of ascorbic acid (e.g., following vitamin supplementation) may introduce a positive bias to the reading. This limitation could be overcome if the patient records the use of vits. C in the application before taking the measurement.

Additionally, bilirubin, a pigment found in urine during certain pathological conditions, typically exhibits a peak absorbance in the visible range (~450 nm). While its absorbance in the 295 nm UV region is lower, highly yellow samples could potentially interfere with the accuracy. Future clinical studies should specifically assess the impact of bilirubin and other chromophores derived from dietary dyes or drug metabolites.

### 3.4. Overall Performance and Applicability

**Figure 6:**
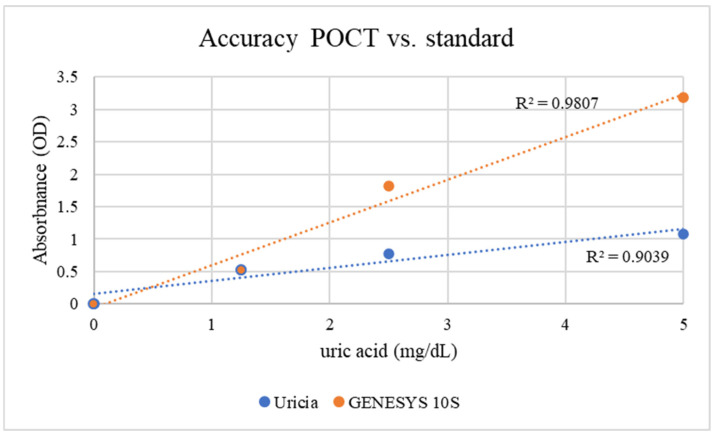
Accuracy of the Uricia POCT device and benchtop spectrophotometer for measuring uric acid in artificial urine (each data point represents the mean of triplicate readings, SD < 0.01).

**Figure 7:**
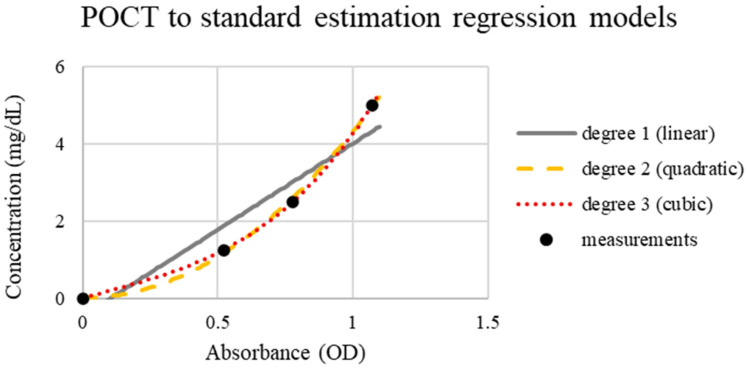
Regression models for estimating the concentration of uric acid from the Uricia device to the standard UV–Vis spectrophotometer (each data point represents the mean of triplicate readings, SD < 0.01).

Uricia and GENESYS 10S revealed strong analytical agreement, as the differences were mainly attributed to optical path discrepancies and detector sensitivity instead of measurement error. The simplified design of Uricia does not compromise the analytical performance (Figure 6). Regression analysis confirmed mild nonlinearity, and cubic modeling offered the highest predictive accuracy (R^2^ = 0.98; error < 0.26 mg/dL) (Figure 7). Comparable modeling approaches are frequently required in portable biosensors to adjust for non-ideal optical behavior [21,29].

The limit of detection and quantitation (LOD and LOQ) for Uricia were calculated using a low-end precision approach based on replicate measurements at the lowest non-zero calibration level (1.25 mg/dL). A linear calibration of Uricia response versus concentration was fitted using non-zero standards (*n* = 3), yielding a slope of 0.1424 response units per concentration unit. The lowest non-zero concentration had a standard deviation of 0.0010 mg/dL. The LOD and LOQ were calculated as LOD = 3.3·σ/slope and LOQ = 10·σ/slope, respectively, where σ is the standard deviation, giving an estimated LOD = 0.0232 mg/dL and LOQ = 0.0702 mg/dL.

**Figure 8:**
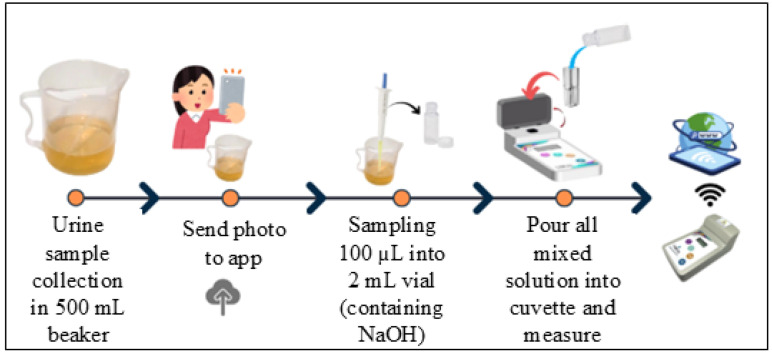
Standard dilution pack.

Although slight deviations were observed at higher uric acid concentrations without NaOH treatment, the calibration model corrected these deviations to linearity. To lengthen the determination interval, a dilution kit was used for this specific sample area (Figure 8). This enabled samples exceeding the saturation threshold to be diluted and automatically corrected to ensure high-concentration measurements [21,22,23].

### 3.5. Cloud Data and Digital Integration

**Figure 9:**
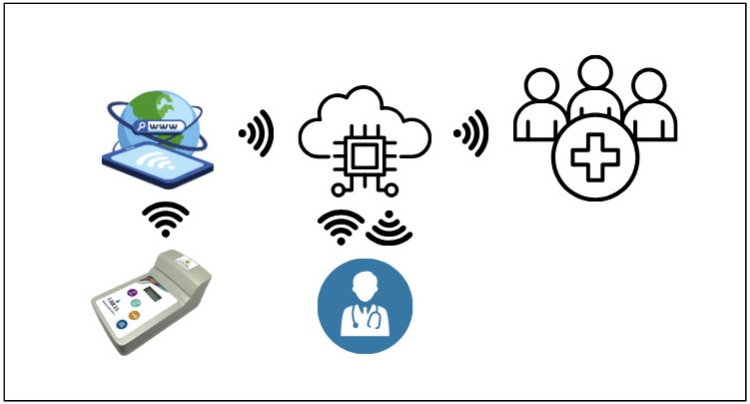
Cloud-based connectivity of the Uricia device (Supplementary Material).

In addition to optical performance, Uricia integrates wireless data transfer and cloud-based analytics on the Internet of Medical Things (IoMT) platform (Figure 9). Data storage, trend monitoring, and integration with electronic medical records were backed by the HIPAA-compliant cloud server. Furthermore, the platform includes predictive models, such as mixed-effects models and machine learning regression models, to predict 24 h uric acid excretion from spot urine values. These mechanisms account for hydration, diurnal changes, and inter-subject variability in physiology. These integrated digital–analytical systems are gaining widespread adoption in next-generation biosensors, such as wearable uric acid monitors and cloud-connected diagnostic tools. The Uricia system is consistent with these trends and provides an effective option for 24 h urine collection—commonly limited by adherence issues—by delivering up-to-date point-of-care estimations with similar laboratory-calculated accuracy [21,29,31,32,33,34,35].

Together, these results position Uricia as a relevant, accessible, and scalable technology within the expanding field of uric acid biosensing, complementing advances across UV-based, SERS, plasmonic, THz, MOF-based, and electrochemical detection platforms [21,22,23,24,25,26,27].

However, it is important to acknowledge the inherent limitations of direct UV spectrophotometry in complex biological matrices. Unlike separation-based methods (HPLC) or enzymatic reactions, UV detection is susceptible to the non-specific absorption of various compounds. Factors such as hydration levels (affecting urine concentration and color depth), dietary compounds, and drug metabolites may alter the optical properties of urine samples. Therefore, Uricia is intended to be a trend-monitoring screening tool rather than a diagnostic confirmation method.

The current predictive framework uses regression models to correlate spot measurements with concentration trends. These models are currently in the developmental phase, and training and validation using real-world patient datasets are planned for upcoming clinical trials.

## 4. Conclusions

This study describes the development and evaluation of Uricia, a portable UV-based device intended to provide rapid measurements of uric acid in urine at a point-of-care. The system relies on fixed-wavelength detection at 295 nm, together with internal calibration routine and optional dilution steps, allowing it to operate in a format that is easier to use than standard laboratory spectrophotometers. Across concentration ranges that could be reliably measured without precipitation, the device produced results that closely followed those of the benchtop GENESYS 10S, particularly when mild alkalinization was used to improve uric acid solubility. The device also performed well in the presence of common urinary constituents, which aligns with the known UV spectral behavior of uric acid.

The addition of a simple dilution kit and secure cloud platform further broadens the range of situations in which the device could be used. These features make it possible to measure samples with elevated concentrations, automatically store the results, and remotely review the data. Early testing of cloud-based analytical tools suggests that spot urine readings can be linked to models capable of estimating 24 h uric acid excretion, a measurement that is normally inconvenient for patients and prone to collection errors. Although predictive algorithms require additional refinement, the initial results indicate that this approach is feasible.

Taken together, these findings suggest that Uricia may offer a practical and affordable alternative for monitoring uric acid in settings where rapid results are needed or access to laboratory equipment is limited. This study focused on the analytical validation of Uricia hardware and algorithms using artificial urine to establish linearity, precision, and limits of detection in a controlled environment. Consequently, the results presented here serve as a foundational proof-of-concept, with extensive clinical trials using patient samples currently underway to validate the performance of the device in real-world scenarios. Although the current study established the accuracy of Uricia device in a controlled artificial matrix to validate the hardware and algorithm, validation in biological samples is a critical next step. Clinical approval to conduct a follow-up study using real patient urine samples will validate Uricia in clinical settings. This will allow for further assessment of how the device handles the complex variability of human urine matrices, examine how the device handles a wider variety of urine matrices, and continue to improve the predictive components of the cloud system. Following these steps, the device shows the potential to be integrated into routine outpatient care or home-based monitoring for patients with hyperuricemia, gout, or related metabolic disorders.

## Figures and Tables

**Table 1:** Absorption peaks of uric acid and potential interferents.

Potential Interfering Compound	Peak (λ nm)
Uric Acid (5 mg/dL)	285
Glucose (250 mg/dL)	197
Ascorbic Acid (50 mg/dL)	246
Albumin (0.5 g/dL)	218
Creatinine (1 mg/dL)	233

## Data Availability

Data used in this research and analysis can be found in the Supplementary Material excel file.

## Supplementary Materials

The following supporting information can be downloaded at: https://www.mdpi.com/article/10.3390/bios16020076/s1, which includes the data used in this research. Additionally, the website, and android app code can be found at https://github.com/tail30/Uricia.git (accessed on 21 January 2026).

## References

[1] Bhole V., de Vera M., Rahman M.M., Krishnan E., Choi H. (2010). Epidemiology of gout in women: Fifty-two–year followup of a prospective cohort. Arthritis Rheum..

[2] Evans P.L., Prior J.A., Belcher J., Hay C.A., Mallen C.D., Roddy E. (2019). Gender-specific risk factors for gout: A systematic review of cohort studies. Adv. Rheumatol..

[3] Wilson L., Saseen J. (2016). Gouty arthritis: A review of acute management and prevention. Pharmacother. J. Hum. Pharmacol. Drug Ther..

[4] Yin H., Liu N., Chen J. (2022). The role of the intestine in the development of hyperuricemia. Front. Immunol..

[5] Martínez-Quintana E., Tugores A., Rodríguez-González F. (2016). Serum uric acid levels and cardiovascular disease: The Gordian knot. J. Thorac. Dis..

[6] Karantas I.D., Miliotou A.N., Siafaka P.I. (2024). An updated review for hyperuricemia and gout management; special focus on the available drug delivery systems and clinical trials. Curr. Med. Chem..

[7] Kimura Y., Tsukui D., Kono H. (2021). Uric acid in inflammation and the pathogenesis of atherosclerosis. Int. J. Mol. Sci..

[8] Borges R.L., Ribeiro A.B., Zanella M.T., Batista M.C. (2010). Uric acid as a factor in the metabolic syndrome. Curr. Hypertens. Rep..

[9] Rai S.K., Burns L.C., De Vera M.A., Haji A., Giustini D., Choi H.K. (2015). The economic burden of gout: A systematic review. Semin. Arthritis Rheum..

[10] Kim J.K., Kim W.J., Hyun J.M., Lee J.S., Kwon J.G., Seo C., Song M.-J., Choi C.W., Hong S.S., Park K. (2017). Salvia plebeia extract inhibits xanthine oxidase activity in vitro and reduces serum uric acid in an animal model of hyperuricemia. Planta Med..

[11] Lipkowitz M.S. (2012). Regulation of uric acid excretion by the kidney. Curr. Rheumatol. Rep..

[12] Rieselbach R.E., Sorensen L.B., Shelp W.D., Steele T.H. (1970). Diminished renal urate secretion per nephron as a basis for primary gout. Ann. Intern. Med..

[13] Kanbara A., Hakoda M., Seyama I. (2010). Urine alkalization facilitates uric acid excretion. Nutr. J..

[14] Kuwabara M., Ae R., Kosami K., Kanbay M., Andres-Hernando A., Hisatome I., Lanaspa M.A. (2025). Current updates and future perspectives in uric acid research, 2024. Hypertens. Res..

[15] Simkin P.A., Hoover P.L., Paxson C.S., Wilson W.F. (1979). Uric acid excretion: Quantitative assessment from spot, midmorning serum and urine samples. Ann. Intern. Med..

[16] John K.A., Cogswell M.E., Campbell N.R., Nowson C.A., Legetic B., Hennis A.J., Patel S.M. (2016). Accuracy and usefulness of select methods for assessing complete collection of 24-hour urine: A systematic review. J. Clin. Hypertens..

[17] Klose S., Stoltz M., Munz E., Portenhauser R. (1978). Determination of uric acid on continuous-flow (AutoAnalyzer II and SMA) systems with a uricase/phenol/4-aminophenazone color test. Clin. Chem..

[18] Morgenstern S., Flor R.V., Kaufman J.H., Klein B. (1966). The Automated Determination of Serum Uric Acid. Clin. Chem..

[19] Slaunwhite W.D., Pachla L.A., Wenke D.C., Kissinger P.T. (1975). Colorimetric, Enzymatic, and Liquid-Chromatographic Methods for Serum Uric Acid Compared. Clin. Chem..

[20] Sakuma R., Nishina T., Kitamura M. (1987). Deproteinizing methods evaluated for determination of uric acid in serum by reversed-phase liquid chromatography with ultraviolet detection. Clin. Chem..

[21] Lin T.-J., Yen K.-T., Chen C.-F., Yan S.-T., Su K.-W., Chiang Y.-L. (2022). Label-Free Uric Acid Estimation of Spot Urine Using Portable Device Based on UV Spectrophotometry. Sensors.

[22] Zhao Y., Wei X., Peng N., Wang J., Jiang Z. (2017). Study of ZnS Nanostructures Based Electrochemical and Photoelectrochemical Biosensors for Uric Acid Detection. Sensors.

[23] Arslan F. (2008). An Amperometric Biosensor for Uric Acid Determination Prepared From Uricase Immobilized in Polyaniline-Polypyrrole Film. Sensors.

[24] RoyChoudhury S., Umasankar Y., Jaller J., Herskovitz I., Mervis J., Darwin E., Hirt P.A., Borda L.J., Lev-Tov H.A., Kirsner R. (2018). Continuous Monitoring of Wound Healing Using a Wearable Enzymatic Uric Acid Biosensor. J. Electrochem. Soc..

[25] Wang Q., Wen X., Kong J. (2020). Recent progress on uric acid detection: A review. Crit. Rev. Anal. Chem..

[26] Ma C., Jiang N., Sun X., Kong L., Liang T., Wei X., Wang P. (2023). Progress in optical sensors-based uric acid detection. Biosens. Bioelectron..

[27] Rocha F.S., Gomes A.J., Lunardi C.N., Kaliaguine S., Patience G.S. (2018). Experimental methods in chemical engineering: Ultraviolet visible spectroscopy—UV-Vis. Can. J. Chem. Eng..

[28] Mahoney E., Kun J., Smieja M., Fang Q. (2019). Point-of-care urinalysis with emerging sensing and imaging technologies. J. Electrochem. Soc..

[29] Norazmi N., Abdul Rasat Z., Mohamad M., Manap H. (2018). UV detection on artificial uric acid using UV-Vis spectrometer. J. Lasers Opt. Photonics.

[30] Brigden M.L., Edgell D., McPherson M., Leadbeater A., Hoag G. (1992). High Incidence of Significant Urinary Ascorbic Acid Concentrations in a West Coast Population—Implications for Routine Urinalysis. Clin. Chem..

[31] Gao Q., Fu J., Xiong F., Wang J., Qin Z., Li S. (2024). A multi-channel urine sensing detection system based on creatinine, uric acid, and pH. Biosensors.

[32] Zhou J.-W., Zheng X.-B., Liu H.-S., Wen B.-Y., Kou Y.-C., Zhang L., Song J.-J., Zhang Y.-J., Li J.-F. (2024). Reliable quantitative detection of uric acid in urine by surface-enhanced Raman spectroscopy with endogenous internal standard. Biosens. Bioelectron..

[33] Onifade O.A., Mohamad Azri D.D., Abu Bakar M.H., Alresheedi M.T., Ng E.K., Mahdi M.A., Muhammad Noor A.S.M. (2025). Surface functionalized plasmonic sensors for uric acid detection with gold-graphene stacked nanocomposites. Photonic Sens..

[34] Mazaheri Z., Federico G., Koral C., Papari G.P., Ullatil L., Russo P., Andreone A.J.S. (2025). Fast Detection of Uric Acid in Urine for Early Diagnosis Using THz Polarized Waves. Sensors.

[35] Ding S., Li S., Lyu Z., Zhou J., Tang S., Fang L., Zang W., Zhang P., Kim S., Li T. (2024). Single-atom materials boosting wearable orthogonal uric acid detection. Med-X.

